# Genetic engineering and raising temperature enhance recombinant protein production with the *cdna1* promoter in *Trichoderma reesei*

**DOI:** 10.1186/s40643-022-00607-2

**Published:** 2022-10-29

**Authors:** Shanshan Jiang, Yue Wang, Qin Liu, Qinqin Zhao, Liwei Gao, Xin Song, Xuezhi Li, Yinbo Qu, Guodong Liu

**Affiliations:** 1 State Key Laboratory of Microbial Technology, Shandong University, 72 Binhai Road, Qingdao, 266237 China; 2 Tobacco Research Institute of Chinese Academy of Agricultural Sciences, 11 Keyuanjingsi Road, Qingdao, 266101 China

**Keywords:** *Trichoderma reesei*, Promoter engineering, Protein expression, Heat induction, β-mannanase

## Abstract

The fungus *Trichoderma reesei* is a powerful host for secreted production of proteins. The promoter of *cdna1* gene, which encodes a small basic protein of unknown function and high expression, is commonly used for constitutive protein production in *T. reesei*. Nevertheless, the production level of proteins driven by this promoter still needs to be improved. Here, we identified that the region 600- to 700-bp upstream of the start codon is critical for the efficiency of the *cdna1* promoter. Increasing the copy number of this region to three improved the production of a heterologous β-mannanase by 37.5%. Screening of several stressful conditions revealed that the *cdna1* promoter is heat inducible. Cultivation at 37 °C significantly enhanced the production of β-mannanase as well as a polygalacturonase with the *cdna1* promoter compared with those at 30 °C. Combing the strategies of promoter engineering, multi-copy gene insertion, and control of cultivation temperature, β-mannanase of 199.85 U/mL and relatively high purity was produced in shake flask, which was 6.6 times higher than that before optimization. Taken together, the results advance the understanding of the widely used *cdna1* promoter and provide effective strategies for enhancing the production of recombinant proteins in *T. reesei*.

**Graphical Abstract:**

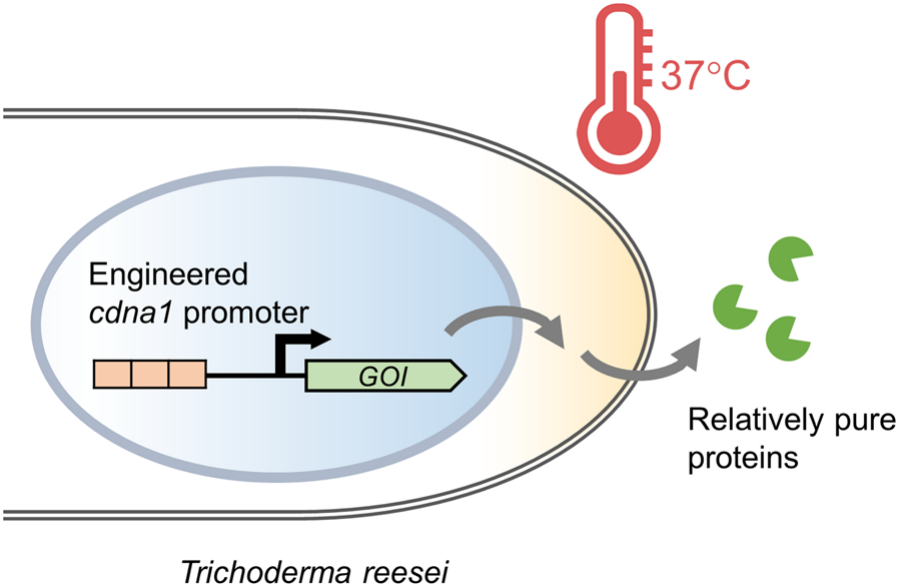

**Supplementary Information:**

The online version contains supplementary material available at 10.1186/s40643-022-00607-2.

## Introduction

The filamentous fungus *Trichoderma reesei* is widely used for cellulase production in industry, and the production level of secreted proteins was reported to be higher than 80 g/L (Bischof et al. 2016; Fonseca et al. 2020). In addition, *T. reesei* has been engineered for the production of other kinds of enzymes and pharmaceutical proteins (e.g., glucoamylase, endo-1,4-beta-xylanase and interferon (Bodie et al. 2021; EFSA Panel on Food Contact Materials et al. 2018; Landowski et al. 2016)). Consequently, the development of a powerful genetic toolbox and efficient fermentation processes are important for using *T. reesei* as a host of protein production.

Strong promoters are essential to achieve high-level production of proteins. The cellulase genes in *T. reesei* are highly inducible by cellulose, sophorose, and lactose, and their promoters have been employed for driving the expression of heterologous proteins (Fitz et al. 2018; Sun et al. 2020; Zou et al. 2012). Because the cellulase inducers can trigger the expression of dozens of endogenous secreted proteins (Herpoël-Gimbert et al. 2008), cellulase gene promoters are unfavorable for downstream purification of target proteins (Chai et al. 2022). In contrast, glucose represses the expression of most extracellular proteins, providing a clean background for secreted protein production using constitutive promoters (Linger et al. 2015; Rantasalo et al. 2019). Li et al. (2012) screened the promoters of 13 carbon metabolic genes and found that *pdc* (encoding pyruvate decarboxylase) and *eno* (encoding enolase) promoters had higher efficiencies in glucose medium. Also, the *cfe1* gene was found to be highly expressed under various conditions, and its promoter was used for gene overexpression in *T. reesei* (Beier et al. 2022). Nevertheless, the efficiencies of constitutive promoters in *T. reesei* are generally much lower than those of cellulase gene promoters (Nakari-Setäläas & Penttilä, 1995).

The gene *cdna1* gene (Trire2_110879; NCBI Gene ID:18,482,212) was identified as one of the most highly expressed genes in glucose medium in *T. reesei* (Nakari et al. 1993). The promoter of *cdna1* was reported to be 20 to 50 times more efficient than that of translation elongation factor 1α gene, *tef1* (Nakari-Setäläas and Penttilä, 1995). This promoter has been used to produce relatively pure proteins in *T. reesei* in glucose-containing media (Sun et al. 2022; Uzbas et al. 2012; Zhang et al. 2022). However, the production level of proteins using the *cdna1* promoter still needs to be improved. In addition, although the *cdna1* promoter is functional in both glucose and cellulose media, whether it is regulated by environmental signals is unclear.

In this study, the characteristics of the *cdna1* promoter was revisited. The key sequence important for its efficiency was identified, which allowed rational sequence engineering for higher gene expression. In addition, the promoter was found to be heat inducible. With the engineered *cdna1* promoter and optimized cultivation condition, the production of a β-mannanase in *T. reesei* was significantly improved.

## Materials and methods

### Strain construction

*T. reesei* QP4, a uracil auxotrophic strain generated from QM9414 by deleting the *pyr4* gene (Zhong et al. 2016), was used as a parent for strain construction in this study. The plasmid pM5p containing the 2,028-bp upstream sequence of *pyr4*, followed by *poman5A* gene (GenBank accession of protein sequence: EPS31069.1) and its 500-bp downstream sequence from *P. oxalicum*, *pyrG* gene from *Aspergillus nidulans* as a selection marker, and the 2,183-bp downstream sequence of *pyr4*, was used for constructing gene expression cassettes (Additional file 1: Fig. S1). The ClonExpress II One Step Cloning Kit (Vazyme, China) was used to generate recombinant plasmids.

The *cdna1* promoters of different lengths, as well as the key sequence fragments (for PcMC) or 5'-UTR of *cel5A* (for MCU5), were amplified from the genomic DNA of QP4 and then inserted into the restriction site *Eco*72I on pM5p. The MCU7 promoter was constructed using fusion and nested PCR, and cloned into the *Eco*72I site on pM5p. The obtained plasmids were digested with restriction enzyme *Pme*I, and the *poman5A* gene expression cassettes carrying homologous arms of *pyr4* were recovered by purification. To construct *poman5A* randomly inserted strains, the PcMC-*poman5A*-*pyrG* fragment was cloned into the *Sac*I and *Eco*RI sites of plasmid pUC19, and then the generated plasmid was linearized with restriction enzyme *Nde*I. The obtained cassettes were transformed into the protoplasts of QP4 as described by Penttilä et al. 1987. Transformants were screened and purified on minimal medium plates, and verified by diagnostic PCR. All the primers used for strain construction were listed in Additional file 1: Table S1.

### Cultivation

All *T. reesei* strains in this study were maintained on Potato Dextrose Agar plates at 30 °C for 7 days for conidiation. Conidia were harvested by washing the plates with normal saline containing 0.01% (w/v) Tween 80. For protein production, biomass measurement, and RNA extraction, fresh conidia were inoculated into 50 mL seed medium at a final concentration of 10^6^/mL, and the Erlenmeyer flasks were incubated in a rotary shaker at 200 rpm at 30 °C for 24 h. Then, 5 mL seed culture was inoculated to 50 mL fermentation medium for continued cultivation at 30 °C unless otherwise indicated.

The seed medium contained (g/L): glucose 20.0, (NH_4_)_2_SO_4_ 5.0, KH_2_PO_4_ 15.0, MgSO_4_·7H_2_O 0.6, CaCl_2_ 0.6, peptone 2.0, FeSO_4_·7H_2_O 0.005, MnSO_4_·H_2_O 0.0016, ZnSO_4_·7H_2_O 0.0014, and CoCl_2_·6H_2_O 0.002. The fermentation medium contained (g/L): glucose 20.0, (NH_4_)_2_SO_4_ 5.0, KH_2_PO_4_ 5.0, MgSO_4_·7H_2_O 0.6, CaCl_2_ 1.0, and yeast extract 20.0. For xylose fermentation medium, glucose was replaced by 20 g/L xylose. For pH 8.0 medium, 50 mM Tris-HCl buffer (pH 8.0) was used to prepare the medium. For low phosphate fermentation medium, the concentration of KH_2_PO_4_ was changed to 0.1 g/L and 50 mM sodium citrate buffer (pH 5.0) was used for medium preparation. Uracil with a final concentration of 1 g/L was added to the medium for the cultivation of QP4.

### Enzyme assays and SDS-PAGE

For β-mannanase activity measurement, the crude enzyme solution was diluted to 500 μL using 0.2 M acetic acid–sodium acetate buffer (pH 4.8) and then mixed with 1.5 mL of 1% (w/v) locust bean gum (Sigma-Aldrich). The reaction system was incubated at 50 °C for 30 min and ended with the addition of 3 mL 3,5-dinitrosalicylic acid reagent. After boiling for 10 min and the addition of 20 mL distilled water, absorbance at 540 nm was determined. The concentration of released product was calculated according to the standard curve of mannose. One unit of β-mannanase activity was defined as the amount of enzyme required to release 1 μmol mannose from the substrate per minute under the above condition. Polygalacturonase activity was measured using the same method except that the substrate was replaced by 1% (w/v) polygalacturonic acid (Sigma-Aldrich). The culture supernatants were mixed with 5 × SDS-PAGE loading buffer (GenStar, China), boiled for 10 min, and centrifuged at 8,000 rpm for 1 min. Equal volumes (30 µl) of samples were loaded into 12.5% (w/v) SDS-PAGE gels for electrophoresis at 120 V for 1 h. Gels were stained with Coomassie Blue R-250 solution for 1 h and then washed with destaining solution (1:1:8 ethanol/acetic acid/water, v/v/v) until a clear background was obtained.

### Biomass measurement

The weight of filter paper was recorded after drying to constant weight. Mycelia were harvested on filter paper by vacuum filtration and then placed in a 65 °C oven to dry to constant weight for weight measurement.

### Nucleic acid extraction and quantitative PCR (qPCR)

The mycelia were collected on filter paper by vacuum filtration and then ground to powder in liquid nitrogen. DNA was extracted using the phenol/chloroform/isoamyl alcohol method as described by Ries et al. (Ries et al. 2014). Total RNA extraction and cDNA synthesis were performed using the RNAiso Reagent (TaKaRa, Japan) and HiScript III RT SuperMix for qPCR (+ gDNA wiper) (Vazyme, China), respectively, according to the manufacturers’ instructions.

For qPCR, diluted genomic DNA or cDNA samples were mixed with primers and TB Green *Premix Ex Taq* II (Tli RNaseH Plus) (TaKaRa, Japan) according to the manufacturer’s instructions. qPCR analysis was performed on LightCycler 480 II system (Roche) using the previously described conditions except setting the annealing temperature at 58 °C (Gao et al. 2017). The 2^−ΔΔ*C*^_*T*_ method was used for data analysis (Livak and Schmittgen 2001). To calculate the copy number of *poman5A*, the mixture of actin gene fragment and *poman5A* gene fragment with a molar ratio of 1:1 was used as a control. To determine relative changes in transcript abundances, the actin gene was used as a reference gene. The results were similar with those calculated with *sar1* gene as a reference (Steiger et al. 2010). The primers used for qPCR are listed in Additional file 1: Table S2, Supporting Information.

### Statistical analysis

Statistical significance tests of differences between samples were performed by calculating *P* values with one-tailed homoscedastic *t* test in the software Microsoft Office 2010 Excel (Microsoft, USA).

## Results and discussion

### Identification of a key sequence in cdna1 promoter

**Fig. 1:**
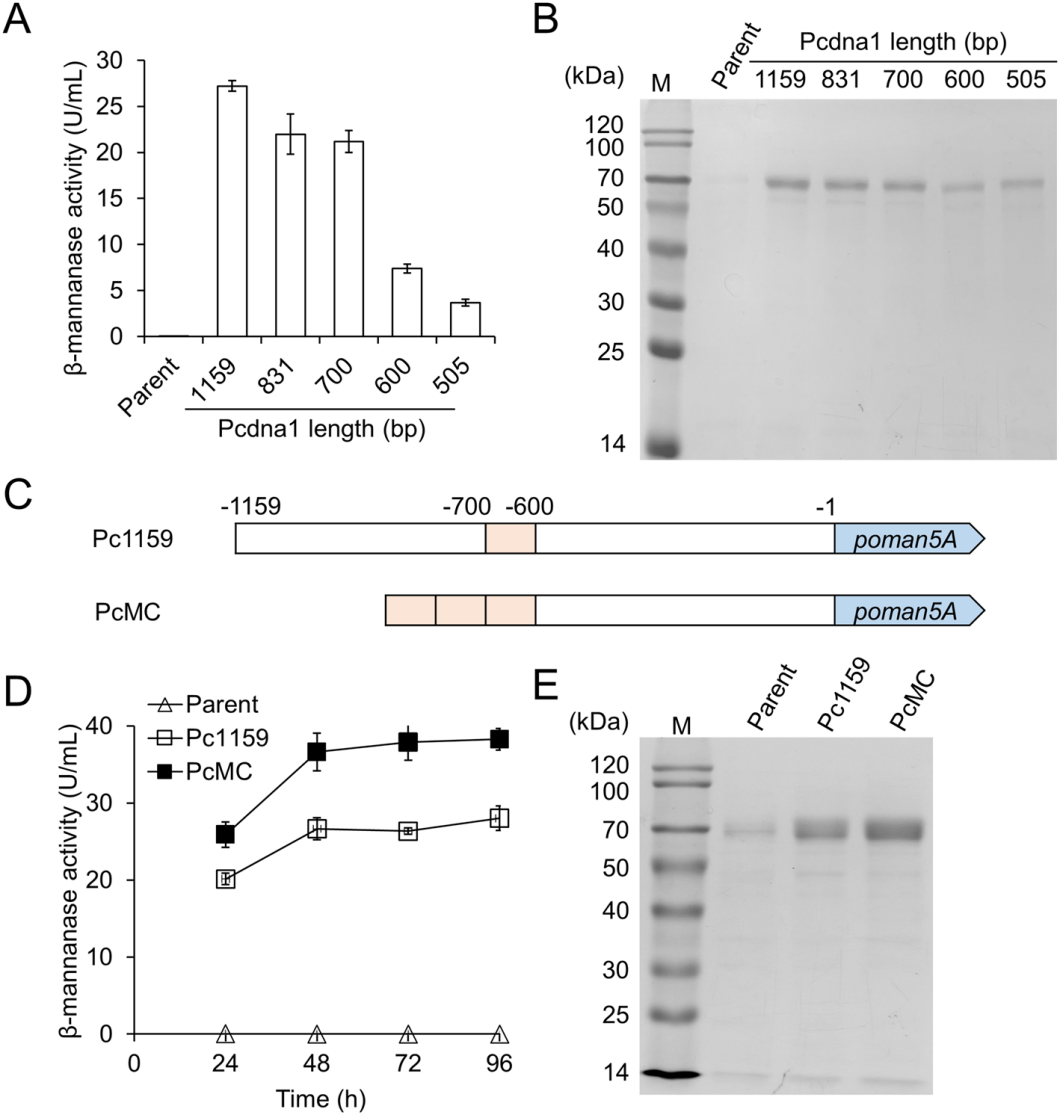
Identification of the key sequence and engineering of *cdna1* promoter. **A** Extracellular β-mannanase activities of strains expressing *poman5A* with different lengths of *cdna1* promoter. The parent strain QP4 was used as a control. The samples were taken after 48 h of cultivation. **B** SDS-PAGE analysis of culture supernatants analyzed in panel A. **C** Schematic representation of the original (Pc1159) and engineered (PcMC) *cdna1* promoter. **D** Extracellular β-mannanase activities of strains expressing *poman5A* with original and engineered *cdna1* promoter. **E** SDS-PAGE analysis of the culture supernatants at 72 h analyzed in panel **D**. Data in panels **A** and **D** represent mean ± SD from triplicate cultivations

In the earliest report, the 1.2-kb sequence preceding the putative start codon of *cdna1* gene was used as the promoter for gene expression (Nakari-Setäläas and Penttilä, 1995). However, the contribution of different regions within this sequence to downstream gene transcription was unknown. We constructed the expression cassettes of β-mannanase gene *poman5A* from *P. oxalicum* driven by the 1,159-bp *cdna1* promoter and its 5′-truncated mutants. The PoMan5A enzyme recombinantly expressed in *Pichia pastoris* was found to be highly stable under high temperature and acidic conditions, making it suitable for many industrial applications (Liao et al. 2014). Each cassette was integrated into the *pyr4* locus of the parent strain QP4 via homologous recombination, and the obtained strains were confirmed to contain only one copy of the *poman5A* gene (Additional file 1: Table S3). The production of β-mannanase using the 831- and 700-bp promoters were about 16% lower than that using the 1,159-bp promoter, while 71.4% and 85.4% declines were observed for the 600-bp and 505-bp promoters, respectively (Fig. 1A). In agree with this, SDS-PAGE analyses of the culture supernatants showed that the protein band of PoMan5A (about 70 kDa) was remarkably lighter in the strains containing 600-bp and 505-bp promoters (Fig. 1B). These results suggested that the sequence between 700- and 600-bp upstream of the start codon was critical for the efficiency of the *cdna1* promoter.

### Increasing the copy number of key sequence improved the efficiency of cdna1 promoter

Increasing the copy number of *cis*-acting activating sequence is an effective strategy for enhancing the efficiency of promoters (Minetoki et al. 1998; Zhang et al. 2018). We constructed the strain expressing *poman5A* using the PcMC promoter containing three copies of the − 700 to − 600 bp sequence (Fig. 1C). Despite the lack of − 1,159 to − 700 bp sequence, the PcMC promoter resulted in 37.4% higher β-mannanase production than the 1,159 bp one at 48 h (Fig. 1D). The improved efficiency of the PcMC promoter was further confirmed by SDS-PAGE of the culture supernatants (Fig. 1E). In the future, detailed mapping of the key activating sequence within the − 700 to − 600 bp region and identification of the bound transcriptional activator(s) are expected to provide more efficient strategies to engineer the *cdna1* promoter.

### Replacing the 5′-untranslated region further improved β-mannanase production

**Fig. 2:**
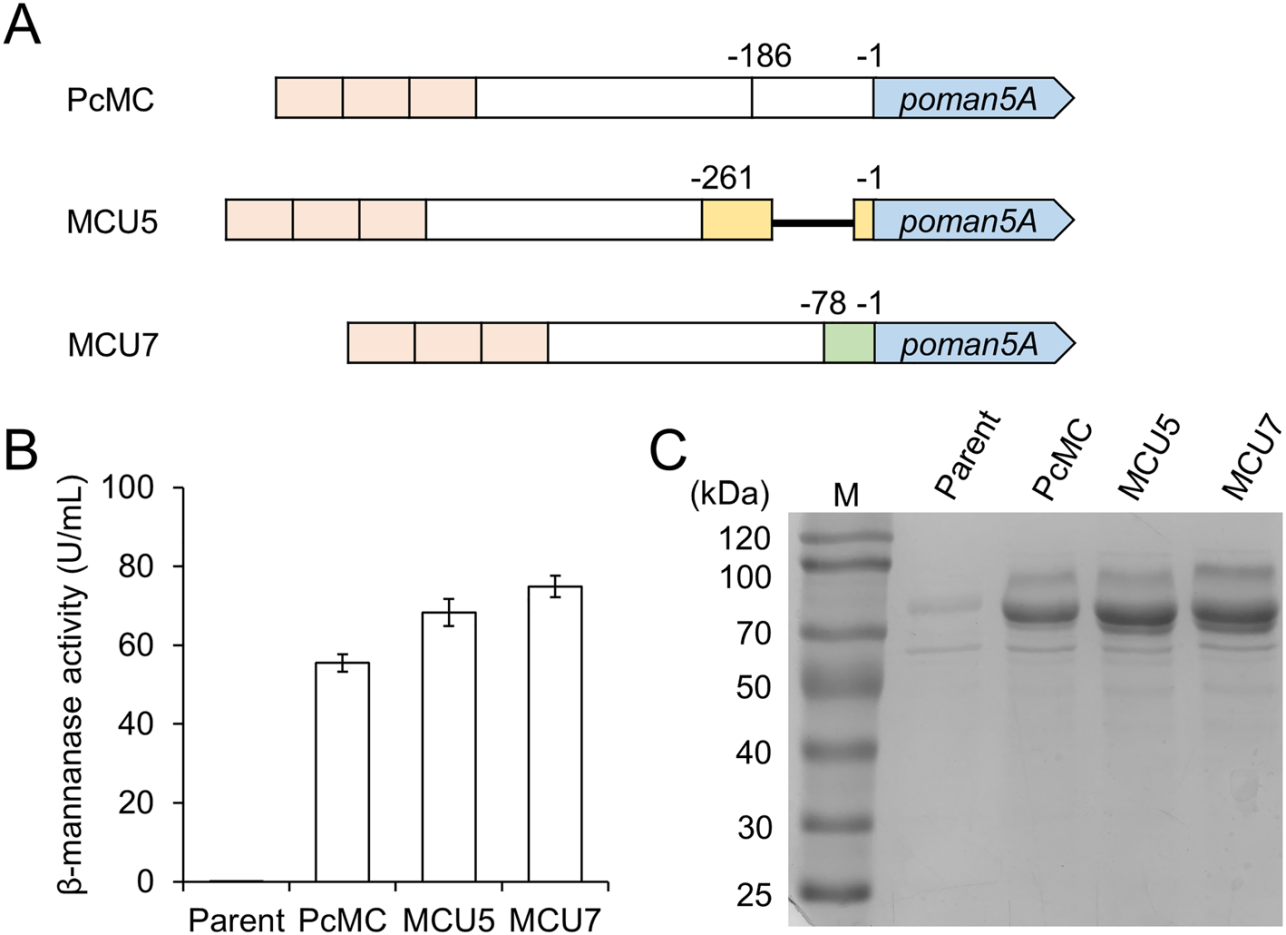
Engineering of the 5′-untranslated region of *cdna1* promoter. **A** Schematic representation of the 5′-UTR replaced promoters. Yellow and green boxes indicate 5′-UTRs from *cel5A* and *cel7A* promoters, respectively. The 123-bp intron in the UTR of *cel5A* is indicated by a thick line. **B** Extracellular β-mannanase activities of strains expressing *poman5A* with different promoters at 48 h. Data represent mean ± SD from triplicate cultivations. **C** SDS-PAGE analysis of the culture supernatants analyzed in panel **B**

Mapping the RNA-seq reads of *T. reesei* (NCBI SRA accession: SRR1057948) to the genomic sequence suggested an about 186-bp 5′-untranslated region (UTR) in the mRNA of *cdna1*. The highly expressed cellulase genes in *T. reesei* contain shorter 5′-UTR sequences than *cdna1* (Saloheimo et al. 1988; Shoemaker et al. 1983), and might contribute to their high production levels. Therefore, we constructed chimeric promoters via replacing the 186-bp 3′ sequence of PcMC by the 5′-UTRs of two major cellulase genes (Fig. 2A). As shown in Fig. 2B and C, replacement of the 5′-UTR in PcMC promoter by those from *cel7A/cbh1* and *cel5A/eg2* improved the production of β-mannanase by 23 and 35%, respectively. The possible efficiency-determining factors in the three 5′-UTRs were compared (Dvir et al. 2013). All three sequences contain adenine at the -3 position, and only that of *cel5A* contains an upstream AUG. We supposed that the different efficiencies of the three 5′-UTRs might be related to their secondary structures.

### Increased temperature induced the expression of genes driven by the cdna1 promoter

**Fig. 3:**
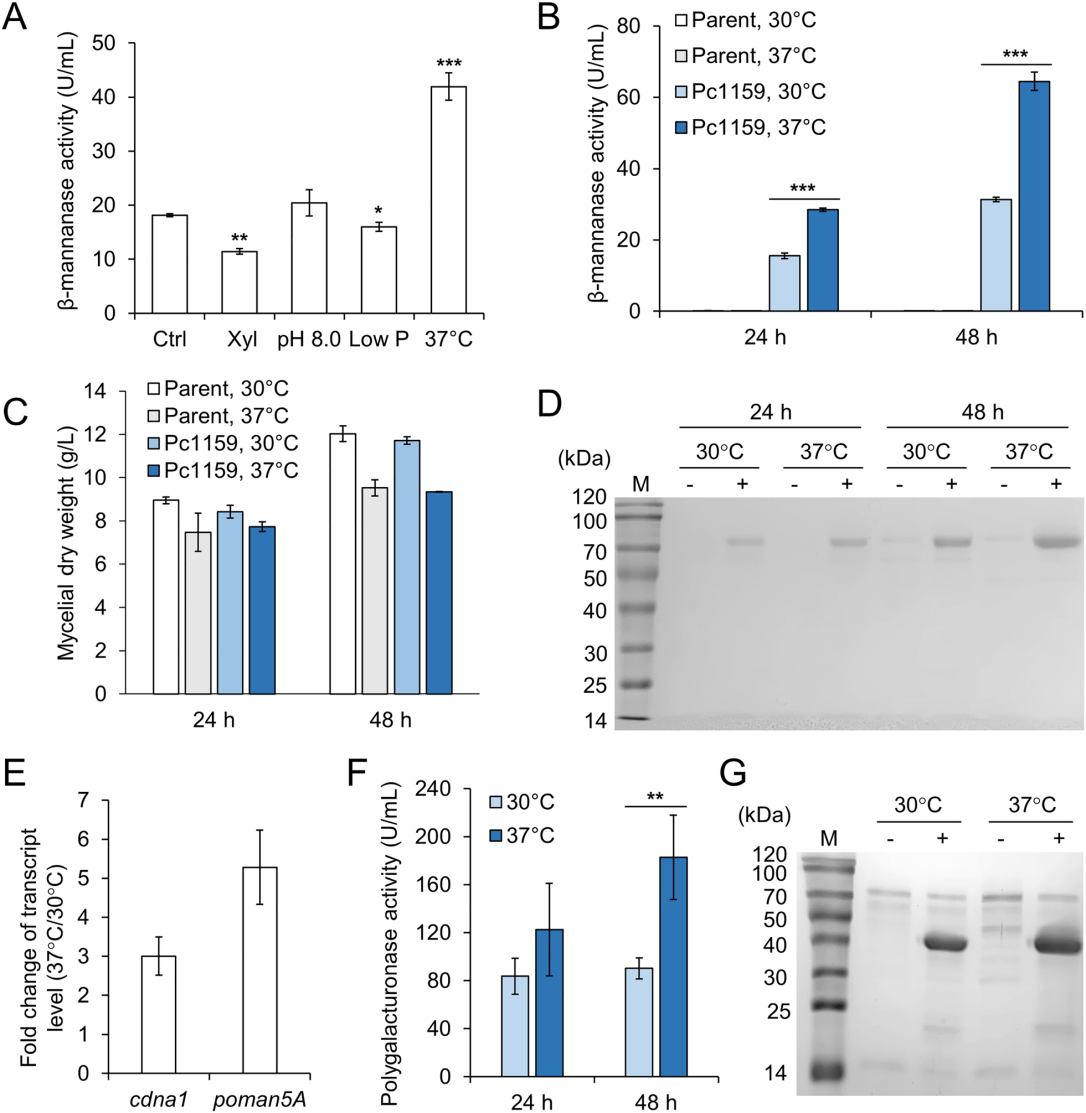
The effect of temperature on protein expression driven by *cdna1* promoter. **A** β-Mannanase production with Pc1159 promoter under different conditions. The samples were taken after 24 h of cultivation. Data represent mean ± SD from duplicate cultivations. Xyl, xylose. Low P, low phosphate. **B** The effect of temperature on β-mannanase production of the strain expressing *poman5A* with Pc1159. The parent strain was used as a control. **C** SDS-PAGE analysis of the culture supernatants analyzed in panel B. **D** The effect of temperature on cell growth. **E** The effect of temperature on the transcript abundances of *cdna1* and *poman5A* in the strain expressing *poman5A* with Pc1159. RNA samples were taken at 24 h. **F** The effect of temperature on endopolygalacturonase production by the strain expressing *popga* with Pc1159. **G** SDS-PAGE analysis of the culture supernatants at 48 h analyzed in panel **F**. Data in panels **B** and **D**–**F** represent mean ± SD from triplicate cultivations. In panel **A**, the statistical significances of the difference between control and cultures under different conditions are shown. In panels B and F, the statistical significances of the difference between cultures at 30 °C and 37 °C are shown. *, *P* < 0.05; **, *P* < 0.01; ***, *P* < 0.001

The *cdna1* gene encodes an 88-amino acid protein of unknown function with a calculated isoelectric point of 11.51. Although *cdna1* is highly transcribed in glucose medium, we speculated that such a small basic protein might be induced under some specific conditions. Therefore, we cultivated pre-grown mycelia of the Pc1159-*poman5A* strain under several different conditions. Cultivation with xylose as sole carbon source, or at pH 8.0, or with low phosphate, resulted in similar or lower β-mannanase production levels. In contrast, cultivation at 37 °C led to remarkably higher β-mannanase production than that at 30 °C (Fig. 3A). Further experiments showed that the increased temperature indeed improved the expression of PoMan5A while inhibiting cell growth (Fig. 3B–D). The abundances of both *cdna1* and *poman5A* were significantly higher at 37 °C relative to 30 °C, indicating that the *cdna1* promoter is heat inducible (Fig. 3E). In addition, the production of a heterologous endopolygalacturonase PoPga (GenBank accession of protein sequence: EPS32977.1, Gao et al. 2022) driven by *cdna1* promoter was also enhanced at 37 °C (Fig. 3F, G), suggesting that elevating the temperature might be a universal strategy for improving protein production with this promoter.

**Fig. 4:**
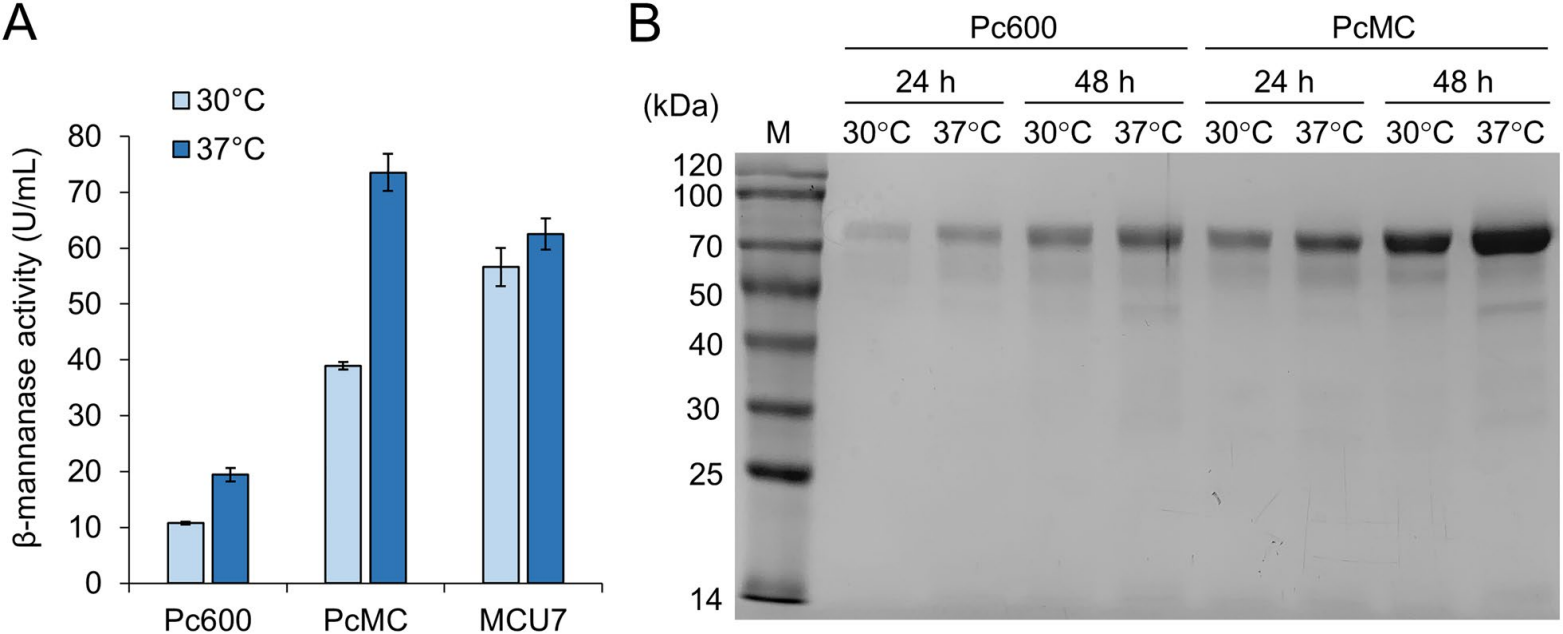
β-Mannanase production by engineered *cdna1* promoters at increased temperature. **A** Extracellular β-mannanase activities of strains expressing *poman5A* with different promoters at 48 h. Data represent mean ± SD from triplicate cultivations. **B** SDS-PAGE analysis of the culture supernatants analyzed in panel **A**

Then, the responses of engineered *cdna1* promoters to elevated temperature were investigated. Despite the decreased strength, the truncated promoter Pc600 maintained the characteristic of heat induction (Fig. 4A). However, the chimeric promoter MCU7 containing *cel7A* 5′-UTR showed similar efficiencies between 30 °C and 37 °C, suggesting that the replaced 186-bp sequence contains the putative heat-responsive element. Cultivation at 37 °C increased β-mannanase production with the PcMC promoter by 89%, which was confirmed by SDS-PAGE analysis of the culture supernatants (Fig. 4B).

### Combined genetic and process engineering enabled high-level production of β-mannanase

**Fig. 5:**
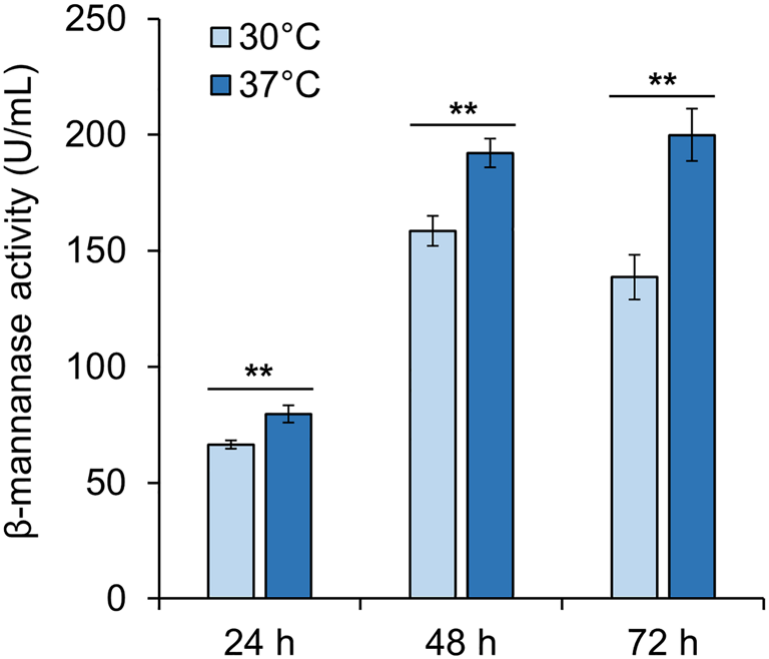
β-Mannanase production by the strain containing multiple PcMC-*poman5A* cassettes. Data represent mean ± SD from triplicate cultivations. The statistical significances of the difference between cultures at 30 °C and 37 °C are shown. *, *P* < 0.05; **, *P* < 0.01; ***, *P* < 0.001

The above results showed that increasing the copy number of -700 to -600 bp sequence, replacement of 5′-UTR, and increasing the cultivation temperature could all enhance the production of β-mannanase. Considering that the 5′-UTR replaced promoters were not heat inducible any more, we constructed strains containing multiple copies of PcMC-*poman5A* gene expression cassettes via random genomic integration. One of the transformants, R-MC-22, was determined to contain two to three copies of the cassettes. The extracellular β-mannanase activity of this strain cultivated at 37 °C reached 199.85 U/ml at 72 h (Fig. 5), which was 6.6 times higher than that integrated with one Pc1159-*poman5A* cassette cultivated at 30 °C (Fig. 1D).

## Conclusions

This study identified the key sequence for the transcription of downstream gene in the *cdna1* promoter of *T. reesei* and found that increasing the cultivation temperature could further enhance the efficiency of this strong promoter. By combining the sequence modification and process improvement, the production level of β-mannanase was significantly enhanced. In the future, elucidation of the regulatory mechanism of the *cdna1* promoter is expected to help the design of more efficient promoters for protein production in *T. reesei*.

### Supplementary Information

**Additional file 1: Table S1.** Primers used for strain construction and verification in this study. **Table S2.** Primers used for qPCR in this study. **Table S3.** Copy numbers of *poman5A* gene in the constructed strains measured by qPCR. **Figure S1. **Map of plasmid pM5p.

## Data Availability

The datasets used and/or analyzed during the current study are available from the corresponding author on reasonable request.

## References

[CR1] Beier S, Stiegler M, Hitzenhammer E, Schmoll M (2022). Screening for genes involved in cellulase regulation by expression under the control of a novel constitutive promoter in *Trichoderma reesei*. Curr Res Biotech.

[CR2] Bischof RH, Ramoni J, Seiboth B (2016). Cellulases and beyond: the first 70 years of the enzyme producer *Trichoderma reesei*. Microb Cell Fact.

[CR3] Bodie E, Virag A, Pratt RJ, Leiva N, Ward M, Dodge T (2021). Reduced viscosity mutants of *Trichoderma reesei* with improved industrial fermentation characteristics. J Ind Microbiol Biotechnol.

[CR4] Chai S, Zhu Z, Tian E, Xiao M, Wang Y, Zou G, Zhou Z (2022). Building a versatile protein production platform using engineered *Trichoderma reesei*. ACS Synth Biol.

[CR5] Dvir S, Velten L, Sharon E, Zeevi D, Carey LB, Weinberger A, Segal E (2013). Deciphering the rules by which 5'-UTR sequences affect protein expression in yeast. Proc Natl Acad Sci USA.

[CR6] Silano V, Barat Baviera JM, Bolognesi C, Brüschweiler BJ, Cocconcelli PS, Crebelli R, Gott DM, Grob K, Lampi E, Mortensen A, Rivière G, Steffensen IL, Tlustos C, Van Loveren H, Vernis L, Zorn H, Glandorf B, Marcon F, Penninks A, Aguilera J, Aguilera-Gomez M, Andryszkiewicz M, Arcella D, Gomes A, Kovalkovicova N, Liu Y, Chesson A, EFSA Panel on Food Contact Materials, Enzymes and Processing Aids (CEP) (2018). Safety evaluation of the food enzyme endo-1,4-beta-xylanase from a genetically modified *Trichoderma reesei* (strain DP-Nzd22). EFSA J.

[CR7] Fitz E, Wanka F, Seiboth B (2018). The promoter toolbox for recombinant gene expression in *Trichoderma reesei*. Front Bioeng Biotechnol.

[CR8] Fonseca LM, Parreiras LS, Murakami MT (2020). Rational engineering of the *Trichoderma reesei* RUT-C30 strain into an industrially relevant platform for cellulase production. Biotechnol Biofuels.

[CR9] Gao L, Li Z, Xia C, Qu Y, Liu M, Yang P, Yu L, Song X (2017). Combining manipulation of transcription factors and overexpression of the target genes to enhance lignocellulolytic enzyme production in *Penicillium oxalicum*. Biotechnol Biofuels.

[CR10] Gao L, Liu G, Zhao Q, Xiao Z, Sun W, Hao X, Liu X, Zhang Z, Zhang P (2022). Customized optimization of lignocellulolytic enzyme cocktails for efficient conversion of pectin-rich biomass residues. Carbohydr Polym.

[CR11] Herpoël-Gimbert I, Margeot A, Dolla A, Jan G, Mollé D, Lignon S, Mathis H, Sigoillot JC, Monot F, Asther M (2008). Comparative secretome analyses of two *Trichoderma reesei* RUT-C30 and CL847 hypersecretory strains. Biotechnol Biofuels.

[CR12] Landowski CP, Mustalahti E, Wahl R, Croute L, Sivasiddarthan D, Westerholm-Parvinen A, Sommer B, Ostermeier C, Helk B, Saarinen J, Saloheimo M (2016). Enabling low cost biopharmaceuticals: high level interferon alpha-2b production in *Trichoderma reesei*. Microb Cell Fact.

[CR13] Li J, Wang J, Wang S, Xing M, Yu S, Liu G (2012). Achieving efficient protein expression in *Trichoderma reesei* by using strong constitutive promoters. Microb Cell Fact.

[CR14] Liao H, Li S, Zheng H, Wei Z, Liu D, Raza W, Shen Q, Xu Y (2014). A new acidophilic thermostable endo-1,4-beta-mannanase from *Penicillium oxalicum* GZ-2: cloning, characterization and functional expression in *Pichia pastoris*. BMC Biotechnol.

[CR15] Linger JG, Taylor LE, Baker JO, Vander Wall T, Hobdey SE, Podkaminer K, Himmel ME, Decker SR (2015). A constitutive expression system for glycosyl hydrolase family 7 cellobiohydrolases in *Hypocrea jecorina*. Biotechnol Biofuels.

[CR16] Livak KJ, Schmittgen TD (2001). Analysis of relative gene expression data using real-time quantitative PCR and the 2^−ΔΔC^_T_ method. Methods.

[CR17] Minetoki T, Kumagai C, Gomi K, Kitamoto K, Takahashi K (1998). Improvement of promoter activity by the introduction of multiple copies of the conserved region III sequence, involved in the efficient expression of *Aspergillus oryzae* amylase-encoding genes. Appl Microbiol Biotechnol.

[CR18] Nakari T, Alatalo E, Penttilä ME (1993). Isolation of *Trichoderma reesei* genes highly expressed on glucose-containing media: characterization of the *tef1* gene encoding translation elongation factor 1 alpha. Gene.

[CR19] Nakari-Setäläas T, Penttilä M (1995). Production of *Trichoderma reesei* cellulases on glucose-containing media. Appl Environ Microbiol.

[CR20] Penttilä M, Nevalainen H, Rättö M, Salminen E, Knowles J (1987). A versatile transformation system for the cellulolytic filamentous fungus *Trichoderma reesei*. Gene.

[CR21] Rantasalo A, Vitikainen M, Paasikallio T, Jäntti J, Landowski CP, Mojzita D (2019). Novel genetic tools that enable highly pure protein production in *Trichoderma reesei*. Sci Rep.

[CR22] Ries L, Belshaw NJ, Ilmén M, Penttilä ME, Alapuranen M, Archer DB (2014). The role of CRE1 in nucleosome positioning within the *cbh1* promoter and coding regions of *Trichoderma reesei*. Appl Microbiol Biotechnol.

[CR23] Saloheimo M, Lehtovaara P, Penttilä M, Teeri TT, Ståhlberg J, Johansson G, Pettersson G, Claeyssens M, Tomme P, Knowles JK (1988). EGIII, a new endoglucanase from *Trichoderma reesei*: the characterization of both gene and enzyme. Gene.

[CR24] Shoemaker S, Schweickart V, Ladner M, Gelfand D, Kwok S, Myambo K, Innis M (1983). Molecular cloning of exo-cellobiohydrolase I derived from *Trichoderma reesei* strain L27. Nat Biotechnol.

[CR25] Steiger MG, Mach RL, Mach-Aigner AR (2010). An accurate normalization strategy for RT-qPCR in *Hypocrea jecorina* (*Trichoderma reesei*). J Biotechnol.

[CR26] Sun X, Zhang X, Huang H, Wang Y, Tu T, Bai Y, Wang Y, Zhang J, Luo H, Yao B, Su X (2020). Engineering the *cbh1* promoter of *Trichoderma reesei* for enhanced protein production by replacing the binding sites of a transcription repressor ACE1 to those of the activators. J Agric Food Chem.

[CR27] Sun Y, Qian Y, Zhang J, Yao C, Wang Y, Liu H, Zhong Y (2022). Development of a novel expression platform for heterologous protein production via deleting the p53-like regulator Vib1 in *Trichoderma reesei*. Enzyme Microb Technol.

[CR28] Uzbas F, Sezerman U, Hartl L, Kubicek CP, Seiboth B (2012). A homologous production system for *Trichoderma reesei* secreted proteins in a cellulase-free background. Appl Microbiol Biotechnol.

[CR29] Zhang H, Yan JN, Zhang H, Liu TQ, Xu Y, Zhang YY, Li J (2018). Effect of gpd box copy numbers in the *gpdA* promoter of *Aspergillus nidulans* on its transcription efficiency in *Aspergillus niger*. FEMS Microbiol Lett.

[CR30] Zhang J, Li J, Gao L, Waghmare PR, Qu J, Liu G (2022). Expression of a SARS-CoV-2 neutralizing nanobody in *Trichoderma reesei*. Chin J Biotech.

[CR31] Zhong L, Qian Y, Dai M, Zhong Y (2016). Improvement of uracil auxotrophic transformation system in *Trichoderma reesei* QM9414 and overexpression of β-glucosidase. CIESC Journal.

[CR32] Zou G, Shi S, Jiang Y, van den Brink J, de Vries RP, Chen L, Zhang J, Ma L, Wang C, Zhou Z (2012). Construction of a cellulase hyper-expression system in *Trichoderma reesei* by promoter and enzyme engineering. Microb Cell Fact.

