# Supplementary material for: Genetic engineering and raising temperature enhance recombinant protein production with the cdna1 promoter in Trichoderma reesei
Source: Bioresour Bioprocess. 2022 Oct 29;9(1):113. doi: 10.1186/s40643-022-00607-2 (PMC10991654; doi:10.1186/s40643-022-00607-2)
Supplement: Supplementary file 1 — Additional file 1: Table S1. Primers used for strain construction and verification in this study. Table S2. Primers used for qPCR in this study. Table S3. Copy numbers of poman5A gene in the constructed strains measured by qPCR. Figure S1. Map of plasmid pM5p. [file 40643_2022_607_MOESM1_ESM.docx]

Supporting Information

**Genetic engineering and raising temperature enhance recombinant protein production with the *cdna1* promoter in *Trichoderma reesei***

**Shanshan Jiang^1^, Yue Wang^1^, Qin Liu^1^, Qinqin Zhao^1^, Liwei Gao^2*^, Xin Song^1^, Xuezhi Li^1^, Yinbo Qu^1^, Guodong Liu^1*^**

^1^ State Key Laboratory of Microbial Technology, Shandong University, 72 Binhai Road, 266237 Qingdao, China

^2^ Tobacco Research Institute of Chinese Academy of Agricultural Sciences, 11 Keyuanjingsi Road, 266101 Qingdao, China

***Correspondence**: gdliu@sdu.edu.cn (G. Liu), gaoliwei01@caas.cn (L. Gao).

**
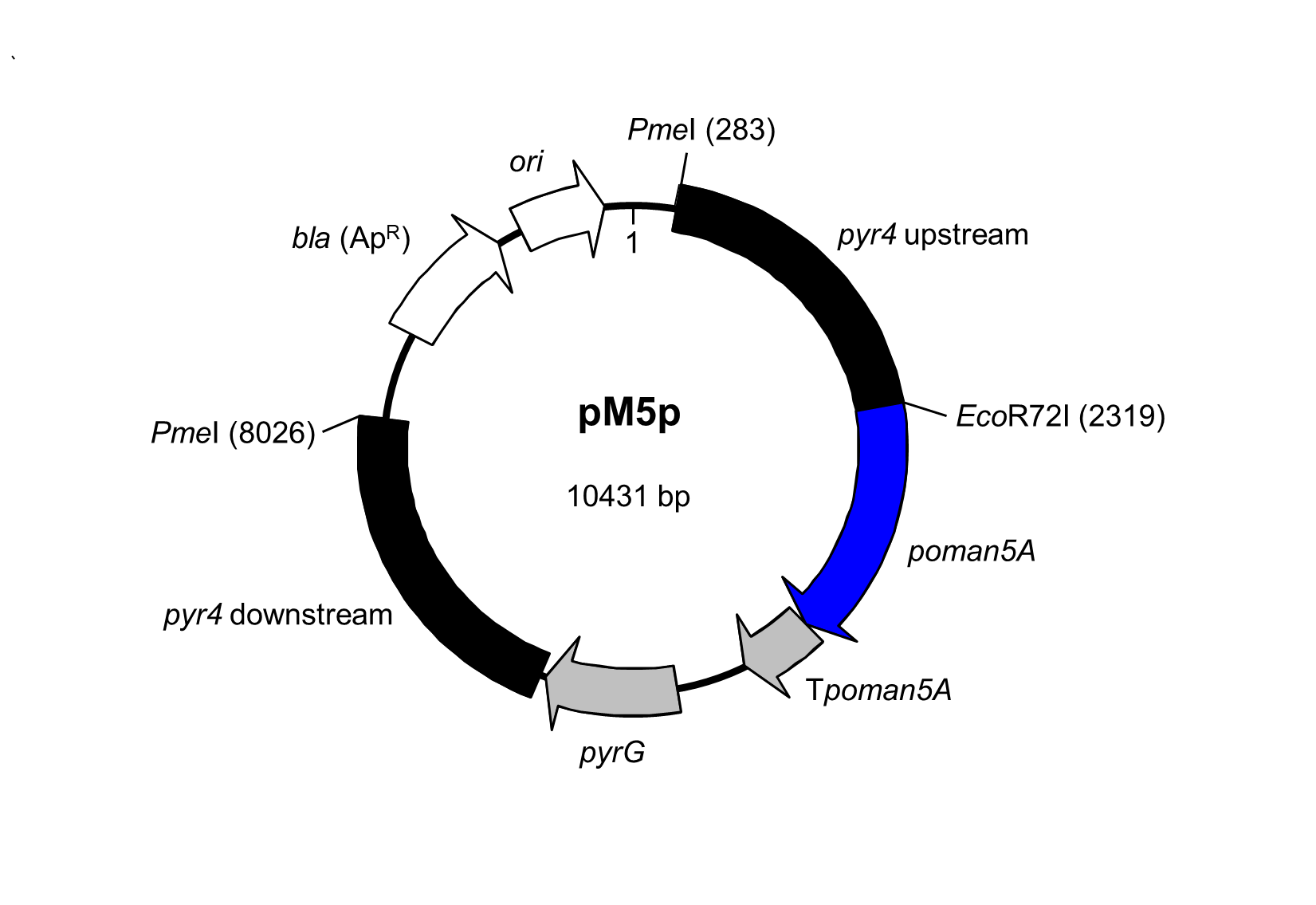
**

**Figure S1.** Map of plasmid pM5p.

**Table S1.** Primers used for strain construction and verification in this study.

| **Primer name** | **Sequence (5′ to 3′)** | **Note** |
| --- | --- | --- |
| Pcdna1-FY | CTCATTACTACCCTCTCTCGGTCTGAAGGACGTGGAATG | Amplification of 1159 bp *cdna1* promoter |
| Pcdna1-RY | GCCGATGAGTACAACATGTTGAGAGAAGTTGTTGGATT | Amplification of 1159 bp, 831 bp, 700 bp, 600 bp, and 505 bp *cdna1* promoter |
| Pcdna1-F831 | CTCATTACTACCCTCTCGGCACCGGACCTCAGCAAATC | Amplification of 831 bp *cdna1* promoter |
| Pcdna1-F700 | ATTACTACCCTCTCCACGTGCGGATCCGGCCCATGTTCTGCCT | Amplification of 700 bp *cdna1* promoter |
| Pcdna1-F600 | ACTACCCTCTCCACGTGCCCAAAACAAGCAACCTTGAACCC | Amplification of 600 bp *cdna1* promoter |
| Pcdna1-F505 | CTCATTACTACCCTCTCGTAGGGCCAGCCAATTAGCG | Amplification of 505 bp *cdna1* promoter |
| Pcdna1-742-F | CTCATTACTACCCTCTCAGATGTCAAACGATTCTGAC | Amplification of -742 bp to -600 bp region of *cdna1* promoter for PcMC construction |
| Pcdna1-742(720)-R | TCCGAAAGGGATGCTCAAGGTACTAGGTGGTGCCACTCCGGAGC |  |
| Pcdna1-720-F | CTCCGGAGTGGCACCACCTAGTACCTTGAGCATCCCTTTC | Amplification of -720 bp to -600 bp region of *cdna1* promoter for PcMC construction |
| Pcdna1-720(700)-R | AGAACATGGGCCGGATCCGAGGTGGTGCCACTCCGGAGCAT |  |
| Pcdna1-700(600)-F | ATGCTCCGGAGTGGCACCACCTCGGATCCGGCCCATGTTCT | Amplification of -700 bp to -1 bp region of *cdna1* promoter for PcMC construction |
| Pcdna1-MC(pyr4-up)-F | CTCATTACTACCCTCTCCACAGATGTCAAACGATTCTGAC | Amplification of PcMC fragment that does not contain the 5'-UTR of *cdna1* for MCU5 construction |
| cdna1-(cel5A-UTR)-R | CCAGGTCAGAAATGGCACTTGTTGATGCTGTGATGATGAAC |  |
| cel5A-UTR(Pcdna1)-F | CATCATCACAGCATCAACAAGTGCCATTTCTGACCTGGATAG | Amplification of the 5'-UTR of *cel5A* for MCU5 construction |
| cel5A-UTR(man)-R | CGCCGATGAGTACAACATCACTGTCGATGACGGGGAGATATTATCG |  |
| cdna1-(cel7A-UTR)-R | GTCTCCTGGAGGATCTGAGTTGCTTGTTGATGCTGTGATGATGAAC | Amplification of PcMC fragment that does not contain the 5'-UTR of *cdna1* for MCU7 construction, paired with Pcdna1-MC(pyr4-up)-F |
| cel7A-UTR(Pcdna1)-F | CATCATCACAGCATCAACAAGCAACTCAGATCCTCCAGGAGACTTG | Amplification of 5'-UTR of *cel7A* and *poman5A* partial sequence from previously constructed plasmid pM5p inserted with P*cbh1* |
| man(h-cel7a)-R | GACGGCCGGAGATGAGATGTAC |  |
| cel7a-UTR(man)-R | ATCGCCGATGAGTACAACATCACGATGCGCAGTCCGCGGTTGAC | Amplification of MCU7 from fusion PCR product, paired with Pcdna1-MC(pyr4-up)-F |
| pyr4-UF(P19) | TTGCCCTCTACGCTGAAAATAG | Located upstream of the *pyr4* upstream arm. Used for the verification and purity test of transformants. |
| man-yz-R(#) | GAGCTGATGCCAGCCCGGCCAACA | Verification of transformants |
| QP4-hph-R1 | TTCTACACAGCCATCGGTCCAGA | Purity test of transformants |
| MC-(P19)-F | GATCCCCGGGTACCGAGCTAGATGTCAAACGATTCTGACC | Amplification of the PcMC-*poman5A*-*pyrG* fragment |
| pyrG-(P19)-R | CGTTGTAAAACGACGGCCAGTGTCAAAGTCCAACTCTTTTC |  |
| Pcdna1-yz-F | GGCACCACCTCCCAAAACAA | Verification of transformants of PcMC-*poman5A*-*pyrG* fragment random insertion, paired with man-yz-R(#) |

**Table S2.** Primers used for qPCR in this study.

| **Primer name** | **Sequence (5′ to 3′)** | **Note** |
| --- | --- | --- |
| man-F | CTCATCGGCGATCTTGCT | Amplification of the fragment of *poman5A* for the preparation of *poman5A*-*actin* mixture as control sample |
| man-R | CGACATGATTCGTCACCAG |  |
| actin-F | AACGAGGCATCACGAAGA | Amplification of the fragment of *actin* for the preparation of *poman5A*-*actin* mixture as control sample |
| actin-R | TGGAAGGTGGACAGGGAG |  |
| man-qp-F | GTCTTTGCGTGGGAGTTG | Amplification of the fragment of *poman5A* for determining gene copy number |
| man-qp-R | CGGGTCGAGTGATTTGAT |  |
| actin-qp-F | AGCCTTCTGTCCTGGGTC | Amplification of the fragment of *actin* for determining gene copy number and transcription analysis |
| actin-qp-R | GCCGTAGAGGTCCTTTCG |  |
| man-qPCR-F | CGGGTAAGCCCTGCTTGTTTGAGG | Amplification of the fragment of *poman5A* for transcription analysis |
| man-qPCR-R | CGACATGATTCGTCACCAGGCAGG |  |
| cdna1-qPCR-F | GACGCTCATTCGAGCACCAGC | Amplification of the fragment of *cdna1* for transcription analysis |
| cdna1-qPCR-R | CCTGTGGTTGTACGTACGTGAC |  |

**Table S3.** Copy numbers of *poman5A* gene in the constructed strains measured by qPCR.

| **Strains** | **Copy number** |
| --- | --- |
| Pc1159-*poman5A* | 1.03 |
| Pc831-*poman5A* | 0.98 |
| Pc700-*poman5A* | 0.86 |
| Pc600-*poman5A* | 0.84 |
| Pc505-*poman5A* | 1.05 |
| PcMC-*poman5A* | 1.14 |
| MCU5-*poman5A* | 0.91 |
| MCU7-*poman5A* | 0.91 |
| R-MC-22 | 2.55 |

**Supplementary data**

The sequence of Pc1159:

TCGGTCTGAAGGACGTGGAATGATGGACTTAATGACAAGAGTTGCCTGGCTATTGAGCTCTGGTACATGGATCTCGAACTGAGAGCGTACAAGTTACATGTAGTAAATCTAGTAGATCTCGCTGAAAGCCCTCTTTCCCGGTAGAAACACCACCAGCGTCCCGTAGGACAAGATCCTGTCGATCTGAGCACATGAATTGCTTCCCTGGATCTGGCGCTGCATCTGTTTCCCCAGACAATGATGGTAGCAGCGCATGGAAGAACCCGGTTGTTCGGAATGTCCTTGTGCTAACAGTGGCATGATTTTACGTTGCGGCTCATCTCGCCTTGGCACCGGACCTCAGCAAATCTTGTCACAACAGCAATCTCAAACAGCCTCATGGTTCCCAGATTCCCTGATTCAGAACTCTAGAGCGGCAGATGTCAAACGATTCTGACCTAGTACCTTGAGCATCCCTTTCGGATCCGGCCCATGTTCTGCCTGCCCTTCTGAGCACAGCAAACAGCCCAAAAGGCGCCGGCCGATTCCTTTCCCGGGATGCTCCGGAGTGGCACCACCTCCCAAAACAAGCAACCTTGAACCCCCCCCCCAAATCAACTGAAGCGCTCTTCGCCTAACCAGCATAAGCCCCCCCCAGGATCGTTAGGCCAAGTGGTAGGGCCAGCCAATTAGCGAGCGGCCATTTGGAGGTCATGGGCGCAGAATGTCCTGACAGTGGTATGATATTGACTGCCCGGTGTGTGTGGCATCTGGCCATAATCGCAGGCTGAGGCGAGGAAGTCTCGTGAGGATGTCCCGACTTTGACATCATGAGGGAGTGAGAAACTGAAGAGAAGGAAAGCTTCGAAGGTTCGATAAGGGATGATTTGCATGGCGGGCGACAGGATGCGATGGCTCGTTGGGATACATAATGCTTGGGTTGGAAGCGATTCCAGGTCGTCTTTTTTTGGTTCATCATCACAGCATCAACAAGCAACGATACAAGCAATCCACTGAGGATTACCTCTCAACTCAACCACTTTCCAAACCATCTCAACTCCCTAAGATTCTTTCAGTGTATTATCACTAGGATTTTTCCCAAGCCGGCTTCAAAACACACAGATAAACCACCAACTCTACAACCAAAGACTTTTTGATCAATCCAACAACTTCTCTCAAC

The sequence of PcMC (the key sequences were colored):

AGATGTCAAACGATTCTGACCTAGTACCTTGAGCATCCCTTTCGGATCCGGCCCATGTTCTGCCTGCCCTTCTGAGCACAGCAAACAGCCCAAAAGGCGCCGGCCGATTCCTTTCCCGGGATGCTCCGGAGTGGCACCACCTCGGATCCGGCCCATGTTCTGCCTGCCCTTCTGAGCACAGCAAACAGCCCAAAAGGCGCCGGCCGATTCCTTTCCCGGGATGCTCCGGAGTGGCACCACCTAGTACCTTGAGCATCCCTTTCGGATCCGGCCCATGTTCTGCCTGCCCTTCTGAGCACAGCAAACAGCCCAAAAGGCGCCGGCCGATTCCTTTCCCGGGATGCTCCGGAGTGGCACCACCTCCCAAAACAAGCAACCTTGAACCCCCCCCCCAAATCAACTGAAGCGCTCTTCGCCTAACCAGCATAAGCCCCCCCCAGGATCGTTAGGCCAAGTGGTAGGGCCAGCCAATTAGCGAGCGGCCATTTGGAGGTCATGGGCGCAGAATGTCCTGACAGTGGTATGATATTGACTGCCCGGTGTGTGTGGCATCTGGCCATAATCGCAGGCTGAGGCGAGGAAGTCTCGTGAGGATGTCCCGACTTTGACATCATGAGGGAGTGAGAAACTGAAGAGAAGGAAAGCTTCGAAGGTTCGATAAGGGATGATTTGCATGGCGGGCGACAGGATGCGATGGCTCGTTGGGATACATAATGCTTGGGTTGGAAGCGATTCCAGGTCGTCTTTTTTTGGTTCATCATCACAGCATCAACAAGCAACGATACAAGCAATCCACTGAGGATTACCTCTCAACTCAACCACTTTCCAAACCATCTCAACTCCCTAAGATTCTTTCAGTGTATTATCACTAGGATTTTTCCCAAGCCGGCTTCAAAACACACAGATAAACCACCAACTCTACAACCAAAGACTTTTTGATCAATCCAACAACTTCTCTCAAC

The sequence of MCU5 (the 5’UTR sequence of *cel5a* was colored):

AGATGTCAAACGATTCTGACCTAGTACCTTGAGCATCCCTTTCGGATCCGGCCCATGTTCTGCCTGCCCTTCTGAGCACAGCAAACAGCCCAAAAGGCGCCGGCCGATTCCTTTCCCGGGATGCTCCGGAGTGGCACCACCTCGGATCCGGCCCATGTTCTGCCTGCCCTTCTGAGCACAGCAAACAGCCCAAAAGGCGCCGGCCGATTCCTTTCCCGGGATGCTCCGGAGTGGCACCACCTAGTACCTTGAGCATCCCTTTCGGATCCGGCCCATGTTCTGCCTGCCCTTCTGAGCACAGCAAACAGCCCAAAAGGCGCCGGCCGATTCCTTTCCCGGGATGCTCCGGAGTGGCACCACCTCCCAAAACAAGCAACCTTGAACCCCCCCCCCAAATCAACTGAAGCGCTCTTCGCCTAACCAGCATAAGCCCCCCCCAGGATCGTTAGGCCAAGTGGTAGGGCCAGCCAATTAGCGAGCGGCCATTTGGAGGTCATGGGCGCAGAATGTCCTGACAGTGGTATGATATTGACTGCCCGGTGTGTGTGGCATCTGGCCATAATCGCAGGCTGAGGCGAGGAAGTCTCGTGAGGATGTCCCGACTTTGACATCATGAGGGAGTGAGAAACTGAAGAGAAGGAAAGCTTCGAAGGTTCGATAAGGGATGATTTGCATGGCGGGCGACAGGATGCGATGGCTCGTTGGGATACATAATGCTTGGGTTGGAAGCGATTCCAGGTCGTCTTTTTTTGGTTCATCATCACAGCATCAACAAGTGCCATTTCTGACCTGGATAGGTTTTCCTATGGTCATTCCTATAAGAGACACGCTCTTTCGTCGGCCCGTAGATATCAGATTGGTATTCAGTCGCACAGACGAAGgtgagttgatcctccaacatgagttctatgagccccccccttgcccccccccgttcaccttgacctgcaatgagaatcccaccttttacaagagcatcaagccgtattaatggcgctgaatagCCTCTGCTCGATAATATCTCCCCGTCATCGACA

The sequence of MCU7 (the 5’UTR sequence of *cel7a* was colored):

AGATGTCAAACGATTCTGACCTAGTACCTTGAGCATCCCTTTCGGATCCGGCCCATGTTCTGCCTGCCCTTCTGAGCACAGCAAACAGCCCAAAAGGCGCCGGCCGATTCCTTTCCCGGGATGCTCCGGAGTGGCACCACCTCGGATCCGGCCCATGTTCTGCCTGCCCTTCTGAGCACAGCAAACAGCCCAAAAGGCGCCGGCCGATTCCTTTCCCGGGATGCTCCGGAGTGGCACCACCTAGTACCTTGAGCATCCCTTTCGGATCCGGCCCATGTTCTGCCTGCCCTTCTGAGCACAGCAAACAGCCCAAAAGGCGCCGGCCGATTCCTTTCCCGGGATGCTCCGGAGTGGCACCACCTCCCAAAACAAGCAACCTTGAACCCCCCCCCCAAATCAACTGAAGCGCTCTTCGCCTAACCAGCATAAGCCCCCCCCAGGATCGTTAGGCCAAGTGGTAGGGCCAGCCAATTAGCGAGCGGCCATTTGGAGGTCATGGGCGCAGAATGTCCTGACAGTGGTATGATATTGACTGCCCGGTGTGTGTGGCATCTGGCCATAATCGCAGGCTGAGGCGAGGAAGTCTCGTGAGGATGTCCCGACTTTGACATCATGAGGGAGTGAGAAACTGAAGAGAAGGAAAGCTTCGAAGGTTCGATAAGGGATGATTTGCATGGCGGGCGACAGGATGCGATGGCTCGTTGGGATACATAATGCTTGGGTTGGAAGCGATTCCAGGTCGTCTTTTTTTGGTTCATCATCACAGCATCAACAAGCAACTCAGATCCTCCAGGAGACTTGTACACCATCTTTTGAGGCACAGAAACCCAATAGTCAACCGCGGACTGCGCATC
